# Chitosan nanoparticle-mediated co-delivery of shAtg-5 and gefitinib synergistically promoted the efficacy of chemotherapeutics through the modulation of autophagy

**DOI:** 10.1186/s12951-017-0261-x

**Published:** 2017-04-11

**Authors:** Yan Zheng, Chang Su, Liang Zhao, Yijie Shi

**Affiliations:** 1 School of Pharmacy, Jinzhou Medical University, Jinzhou, 121000 People’s Republic of China; 2 School of Veterinary Medicine, Jinzhou Medical University, Jinzhou, 121000 People’s Republic of China

**Keywords:** Autophagy, Apoptosis, shAtg-5, Gefitinib, Nanoparticles

## Abstract

**Background:**

Autophagy reportedly plays vital and complex roles in many diseases. During times of starvation or energy deficiency, autophagy will occur at higher levels to provide cells with the nutrients or energy necessary to survive in stressful conditions. Some anti-cancer drugs induce protective autophagy and reduce cell apoptosis. Autophagy can adversely affect apoptosis, and blocking autophagy will increase the sensitivity of cells to apoptosis signals.

**Methods:**

We designed chitosan nanoparticles (NPs) to promote the co-delivery of gefitinib (an anti-cancer drug) and shRNA-expressing plasmid DNA that targets the Atg-5 gene (shAtg-5) as an autophagy inhibitor to improve anti-cancer effects and autophagy mediation.

**Results:**

The results showed that when compared to treatment with a single drug, chitosan NPs were able to facilitate the intracellular distribution of NPs, and they improved the transfection efficiency of gene in vitro. The co-delivery of gefitinib and shAtg-5 increased cytotoxicity, induced significant apoptosis through the prohibition of autophagy, and markedly inhibited tumor growth in vivo.

**Conclusions:**

The co-delivery of gefitinib/shAtg-5 in chitosan NPs produced superior anti-cancer efficacy via the internalization effect of NPs, while blocking autophagy with shAtg-5 enhanced the synergistic antitumor efficacy of gefitinib.

## Background

Current conventional cancer therapies primarily depended on single chemical drugs that defend against cancer; however, the curative effects of these agents seem to be unsatisfactory, and they often result in chemotherapy failure due to a decline in the patient’s response to drugs—a phenomenon known as acquired drug resistance [1–3]. Therefore, it is necessary to find a new synergistic strategy to strengthen the chemotherapeutic effect of a given agent. It is well known that autophagy plays an important role in the development of cell death. First, as it is an important means of material recycling, and given its involvement in cell homeostasis maintenance, autophagy removes damaged proteins and cell organelles in stress states, thus minimizing the extent of cell damage and maintaining cell stability [4]. In tumor therapy in particular, autophagy has been shown to be one of factors associated with chemotherapy, radiotherapy, and biological immunotherapy tolerance. Therefore, to some extent, the increase in autophagy reduces the cytotoxic and apoptotic effects of anti-cancer drugs. Autophagy can adversely affect apoptosis, and blocking autophagy will increase cell sensitivity to apoptosis signals [5–7].

Nanoparticles (NPs) as nano-scaled carriers showed excellent value and potential for improving drug/gene delivery [8–14]. Owing to their small particle size and high charge potential, NPs depend on enhanced permeability and retention (EPR) effects to achieve massive accumulation of drugs around the tumor while attenuating toxic damage to healthy organs [15–18]. Seeing as both drug molecules and genes can be encapsulated within the core of NPs, safe and effective targeted co-therapies featuring a drug and genes could be successfully achieved to offer synergistic effects against tumor development [19–22].

To confirm whether autophagy regulation plays a synergistic role in enhancing the antitumor efficacy of a chemotherapeutic drug, in this work, we prepared chitosan (CS) NPs with the combined co-delivery of gefitinib and short hairpin (sh)RNA-expressing plasmid DNA targeting the Atg-5 gene (shAtg-5) to improve anti-cancer effects. It was found that free gefitinib and gefitinib NPs induced cell death, while the autophagy effects were also simultaneously enhanced to some extent, which indicated that autophagy might have both positive and negative effects on the induction of cell apoptosis. The introduction of shAtg-5 as an inhibitor of autophagy efficiently downregulated the expression of the autophagy-related protein Atg-5. Furthermore, the cytotoxicity and apoptosis of cells treated with the co-delivery of shAtg-5 and gefitinib loaded in CS NPs were significantly enhanced upon autophagy inhibition.

## Methods

### Materials

CS of medium molecular weight (deacetylation degree, 80%; molecular weight, 400,000) was purchased from Haixin Biological Product Co., Ltd. (Zhejiang, People’s Republic of China). Gefitinib was purchased from Eastbang Pharmaceutical Co., Ltd. (Guangdong, People’s Republic of China). The pGCsi-U6/Neo/GFP-Atg-5 shRNA-expressing plasmid (p)DNA (Atg-5 shRNA, shAtg-5) that targeted the Atg-5 mRNA sequence (TTTCATTCAGAAGCTGTTT), as well as the pGCsi-U6/Neo/GFP-shRNA-expressing pDNA (pEGFP), were purchased from Genechem Co., Ltd. (Shanghai, People’s Republic of China). All of the other purchased chemicals were of analytical grade and were obtained from a variety of vendors. A549 cells and PLC cells were obtained from Jinzhou Medical University (Liaoning, People’s Republic of China).

### Preparation and characterization of gefitinib/shAtg-5 NPs

According to our previous study [23], gefitinib/shAtg-5 NPs were prepared through an electrostatic interaction between positively charged CS and negatively charged sodium tripolyphosphate (TPP). The particle size, zeta potential, and polymer dispersity index (PDI) were determined by dynamic light scattering (Zetasizer Nano ZS; Malvern Instruments, Malvern, UK), and the morphology of NPs was observed by means of a transmission electron microscope (JEM-1200EX; JEOL, Tokyo, Japan). The drug-release pattern was also investigated in vitro. The difference between the amount of the initially added drug and the drug in the supernatant was measured by absorbance detection using an ultraviolet (UV)/Vis spectrophotometer (model 1601; Shimadzu, Kyoto, Japan) to determine the encapsulation efficiency (EE) of the drug in the NPs.

### In vitro transfection experiments

In order to evaluate the enhanced transfection efficiency of genes through their encapsulation in NPs, free enhanced green fluorescent protein (EGFP) as the reporter gene and EGFP-loaded NPs were incubated with cells at a density of 5 × 10^4^ cells/mL for 48 h. After that, the cells were washed with ice-cold phosphate buffered saline (PBS), and they were observed using confocal laser scanning microscopy.

### Cell viability assays

To investigate the regulation of cell apoptosis by autophagy, a 3-(4,5-dimethylthiazol-2-yl)-2,5-diphenyltetrazolium bromide (MTT) assay was adopted to determine cell viability in both PLC cells and A549 cells at a density of 5 × 10^4^ cells/mL. Free gefitinib, free gefitinib and shAtg-5, gefitinib NPs, and gefitinib/shAtg-5 NPs were chosen for incubation with the cells for 24 h at 37 °C under 5% CO_2_. The absorbance of the solution was quantified using a BioTek Syneray-2 microplate reader (BioTek Instruments, Winooski, VT, USA) to measure absorbance at 490 nm.

### Uptake ability of different kinds of NPs in cells

Different kinds of NPs were internalized into cells, and NP distributions were observed using real-time confocal laser scanning microscopy (FluoView FV10i; Olympus Corporation, Tokyo, Japan). NP uptake rates were determined by calculating the fluorescence intensity ratio using a microplate reader (Synery-2; BioTek Instruments). Rhodamine B-labeled NPs were incubated with cells at a density of 5 × 10^4^ cells/mL at 37 °C under 5% CO_2_, and the nuclei were stained with Hochest (blue) for 15 min at 37 °C [24, 25]. At a predetermined time, the cellular distribution of different kinds of NPs was traced with the help of real-time confocal laser scanning microscopy. Basing on our previous study [26], after the cells were treated with fluorescein isothiocyanate (FITC)-labeled NPs, cold PBS was used to wash cells to remove the uninternalized NPs, while quantification of intracellular NPs was detected using a microplate reader by checking the fluorescence of FITC, which is excited at 485 nm and emitted at 528 nm. The relative fluorescence ratio (RFR, %), which represents the relative uptake rates of NPs, was calculated by determining the ratio of the fluorescence intensity of internalized FITC-labeled NPs to that of the initially added FITC-labeled NPs.

### Green fluorescent protein-light chain 3 (GFP-LC3B) plasmid transfection

Cells stably transfected with GFP-LC3 plasmid were seeded into 24-well cell culture plates for incubation for 24 h; the number of cells in each well reached a density of 5 × 10^4^ cells/mL. Cells were treated with free gefitinib and gefitinib-loaded NPs for 24 h. Finally, these cells were washed and GFP-LC3 was able to emit bright green fluorescence by confocal laser scanning microscope.

### Annexin V-FITC/propidium iodide (PI) staining by flow cytometry (FCM)

According to the protocol of our previous study [27], free gefitinib, gefitinib and shAtg-5, gefitinib NPs, and gefitinib/shAtg-5 NPs were chosen to determine the extent of cell apoptosis using the flow cytometer, FACS-Calibur (Becton–Dickinson, Franklin Lakes, NJ, USA).

### Western blot assay

Western blot assay was performed to determine the levels of relative proteins when free gefitinib, gefitinib and shAtg-5, gefitinib NPs, and gefitinib/shAtg-5 NPs were incubated with A549 and PLC cells for 24 h. Briefly, the proteins were transferred to a membrane (typically nitrocellulose or PVDF), where they were stained with antibodies specific to the target protein. Western blotting was performed according to the manufacturer’s instructions (Cell Signaling Technology, Danvers, MA, USA). Protein bands were detected using a gel imaging system (iBox Scientia 600; UVP, LLC., Upland, CA, USA).

### Mice and in vivo tumor studies

Six- to eight-week-old female nude mice (BALB/c nude mice) were purchased from Beijing Vital River Laboratory Animal Technology Company (Beijing, People’s Republic of China). PLC cells (1 × 10^7^ cells/mL) were re-suspended in 100 μL PBS and injected subcutaneously into the anterior flank of 25 mice (five in each group). Two weeks later, when the tumor volume reached ~150 mm^3^, the mice were treated with intraperitoneal injections of PBS, free gefitinib, gefitinib and shAtg-5, gefitinib NPs and gefitinib/shAtg-5 NPs for 5 consecutive days each week for 3 weeks. Tumor diameters were measured twice per week with a caliper, and tumor volumes were calculated using the formula [(width)^2^ × length]/2 (mm^3^). The mice were sacrificed on day 20, the tumors were then isolated, and the tumor specimens were prepared as paraffin-embedded sections for histopathological analysis. All animal studies were conducted according to the regulations for animal experimentation issued by the State Committee of Science and Technology of the People’s Republic of China.

## Results

### Preparation and characterization of gefitinib/shAtg-5 NPs

**Fig. 1:**
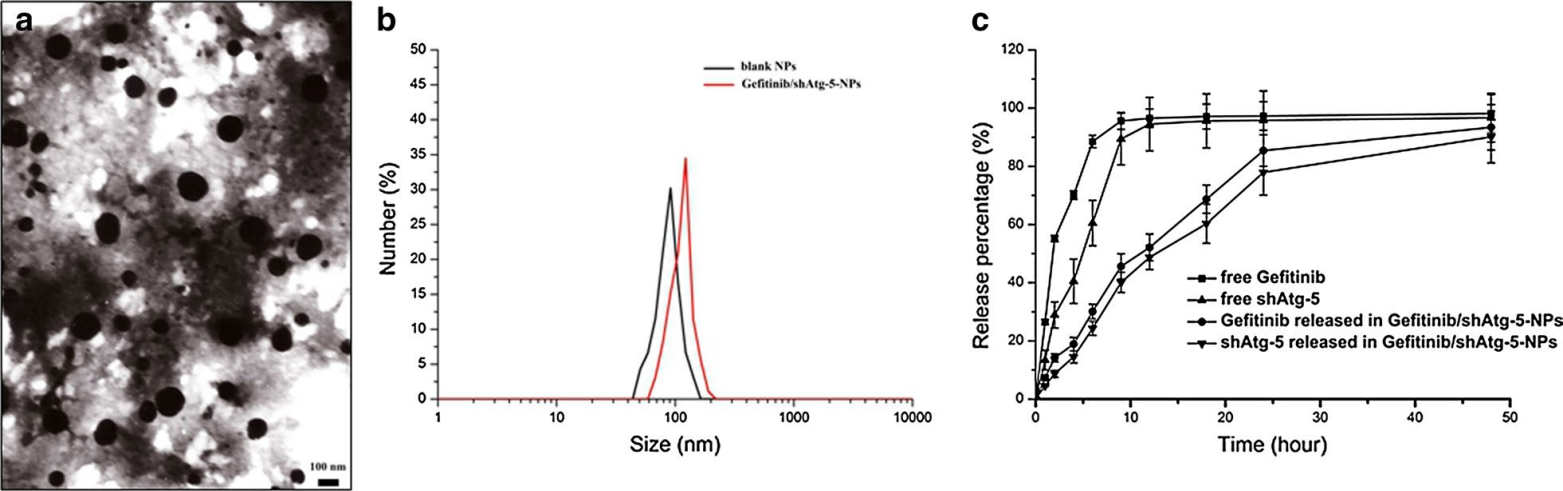
Characterization of gefitinib/shAtg-5 NPs. TEM images of gefitinib/shAtg-5 NPs (**a**), DLS analysis of the obtained gefitinib/shAtg-5 NPs (**b**), and the in vitro release profile of gefitinib/shAtg-5 NPs in phosphate buffered saline (pH 7.4 at 37 °C) for 48 h (**c**). The results were expressed as mean ± SD (n = 3)

Gefitinib/shAtg-5 NPs were prepared using an ion gelation method and they were characterized as having a smaller average particle size at 106.5 ± 2.3 nm; they exhibited a mean positive zeta potential of about 21.2 ± 5.7 mV and a lower PDI of 0.17 ± 0.09. The EEs of gefitinib and shAtg-5 were about 80.5 ± 4.2% and 86.7 ± 4.7%, respectively. The results also showed that when compared with free drugs, the release process of gefitinib and shAtg-5 encapsulated in the NPs could be significantly prolonged; they displayed a biphasic drug-release pattern, which was advantageous for retaining the drugs at high concentrations in the tumor sites for a longer period of time, ultimately improving the anti-tumor efficacy. Judging by the release curve of the drug-loaded NPs, the NPs also controlled the slow and smooth release of gefitinib and shAtg-5; 85.4% of gefitinib and 77.9% of shAtg-5 diffused from the NPs into the medium within 24 h, and more than ~90% of the total gefitinib and shAtg-5 had slowly released from the NPs into the medium within 48 h. On the contrary, over 90% of free gefitinib and shAtg-5 had rapidly entered the PBS medium within 9 h (Fig. 1).

### Gene transfection evaluation in vitro

**Fig. 2:**
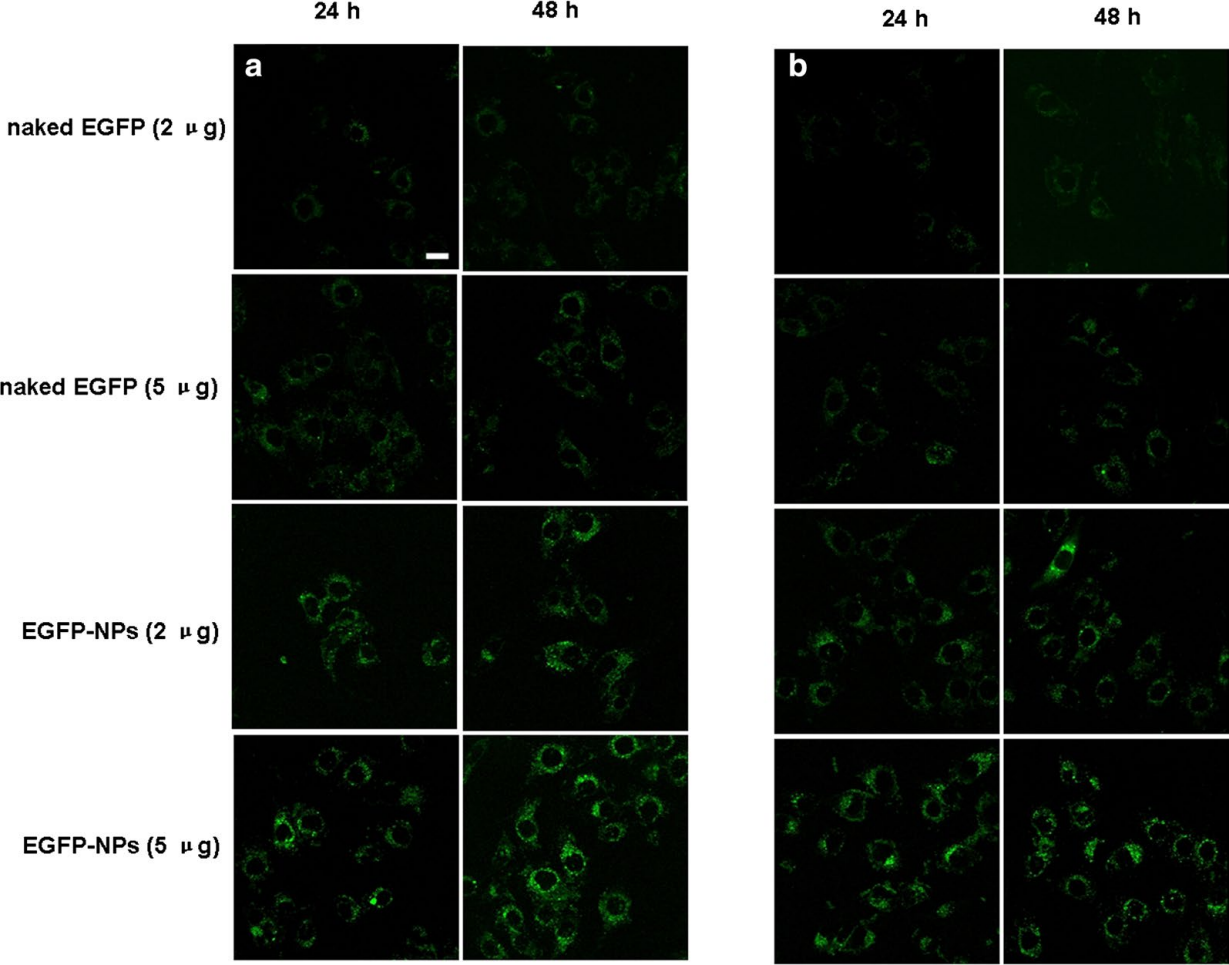
The in vitro transfection efficiency of EGFP-loaded NPs was investigated by observing the microscopic images of A549 cells and PLC cells following incubation with free EGFP and EGFP-loaded NPs for 48 h (**a** A549 cells; **b** PLC cells). *Scale bar* 50 μm

Intracellular gene transfection effects in A549 cells and PLC cells were observed using confocal laser scanning microscopy. The results, as illustrated in Fig. 2, demonstrated that transfected cells showed a green intracellular color following incubation with free EGFP and EGFP-loaded NPs. Conversely, the results indicated that naked plasmid was not easily internalized into the cells, resulting in a weaker green intensity within the cells. In contrast, CS as a potential gene carrier had combined with DNA through an electrostatic attraction between the positively charged amino group of CS and the negatively charged DNA. DNA was efficiently protected from enzymatic degradation by becoming encapsulated in the CS–DNA complex, and the transfection efficiency of DNA was significantly improved within the cells in vitro [28–32]. Plasmid DNA was coated by CS to form the NPs, and it was able to become internalized within the cells, primarily through several endocytic pathways including caveolae-mediated endocytosis, macropinocytosis, and clathrin-mediated endocytosis, thus leading to significant improvements in the in vitro transfection effects. Additionally, gene transfection efficiency was dose dependent and generally increased upon dose augmentation of gene.

### MTT assay

**Fig. 3:**
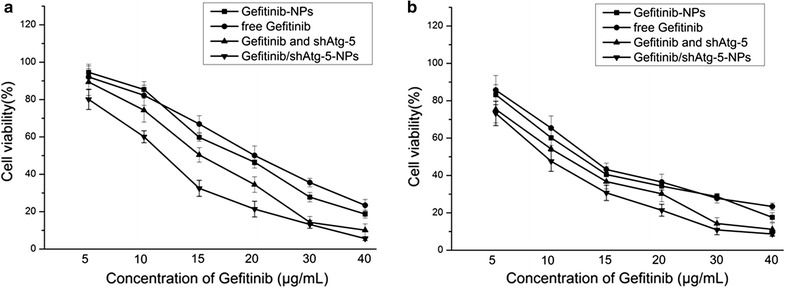
The in vitro viability of A549 cells (**a**) and PLC cells (**b**) treated with various gefitinib formulations. The results were expressed as mean ± SD (n = 3)

The results shown in Fig. 3 demonstrated that when the free drug or the drug-loaded NPs were used to treat the PLC and A549 cells, gefitinib/shAtg-5 NPs showed the lowest cell viability for both cell types; the IC50 values for the gefitinib/shAtg-5 NP-treated A549 and PLC cells were 11.8 and 9.53 μg/mL, respectively, within 24 h. This suggests that the greatest inhibitory effects and the highest cytotoxicity levels could be achieved by the co-delivery of gefitinib and shAtg-5. When shAtg-5 acts as a mediator to silence the Atg-5 protein, the autophagy effects were significantly inhibited and the cells’ sensitivity toward the drug was further enhanced, leading to greater apoptosis. The synergistic effects between autophagy and apoptosis were also found to be negatively correlated. When cells were incubated with free gefitinib, the IC50 values for the treated A549 and PLC cells were 20.1 and 13.4 μg/mL, respectively, within 24 h. Similarly, after being treated with gefitinib and shAtg-5, the IC50 values were reduced to 15.1 μg/mL for A549 cells and to 11.2 μg/mL for PLC cells. The results further confirmed that preventing the development and progression of autophagy could accelerate cell apoptosis. In addition, drug-loaded CS NPs facilitated intracellular uptake through electrostatic attraction, as CS NPs carrying positive charges tended to combine with the negatively charged cell-surface proteins. The IC50 values (at 24 h) of the A549 and PLC cells treated with gefitinib NPs were 18.7 and 12.5 μg/mL, respectively, within 24 h.

### Distribution of NPs in cells

**Fig. 4:**
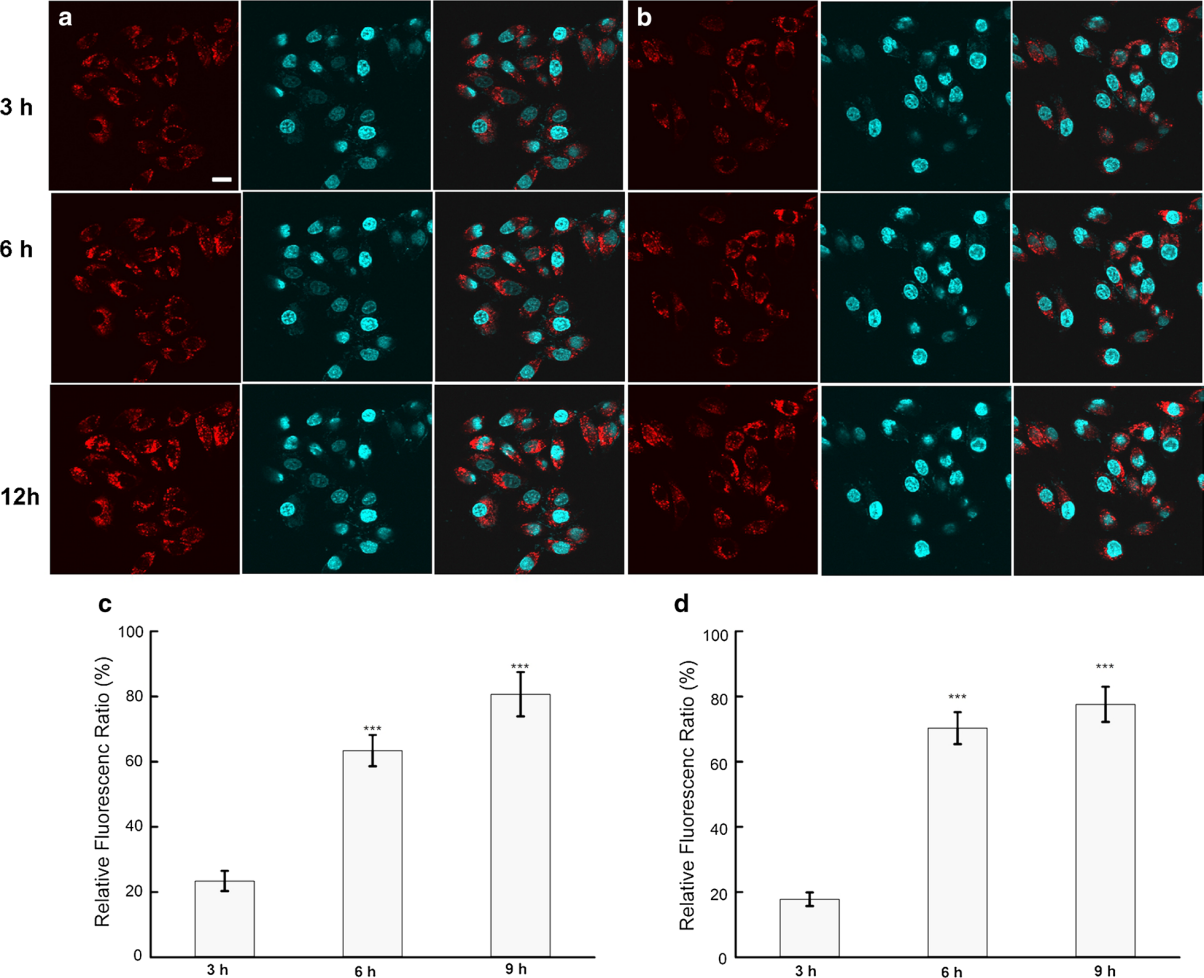
The uptake of NPs in PLC cells and A549 cells. Fluorescence microscopy analysis of the uptake of Rhodamine B-labeled NPs in PLC cells (**a**) and A549 cells (**b**). *Scale bar* was 50 μm. Fluorescence spectrum analysis of the uptake of FITC-labeled NPs in PLC cells (**c**) and A549 cells (**d**). The results were expressed as mean ± SD (n = 3). ***p < 0.001 versus the NP-treated cells within 3 h

The uptake of NPs in PLC cells and A549 cells was observed, as shown in Fig. 4. Rhodamine B, as a fluorescent marker, was encapsulated in the NPs and the nuclei were stained with Hoechst (blue) for 15 min at 37 °C. The results demonstrated that the NPs had attached to the surface of the cell membrane, as represented by the appearance of some weak fluorescent red dots around the cells within the initial 3 h. With the passage of time, more red spots began to move toward the cells’ interior, where they sprinkled throughout the entire cytoplasm. The fluorescence intensity within the cells was also quantified by a microplate reader and the uptake ratio of the NPs was represented by the relative fluorescence ratio (RFR, %). This suggested that about 20% of the total amount of NPs had internalized into both cell types within the first 3 h, and the uptake ratio had increased to 70.3% at 6 h and to 77.6% at 9h in the A549 cells. Similarly, about 63.4% of all NPs were internalized into the PLC cells within 6 h and 80.7% had become internalized within 9 h.

### Confocal microscope images of GFP-LC3-transfected A549 cells and PLC cells treated with free gefitinib and gefitinib-loaded NPs

**Fig. 5:**
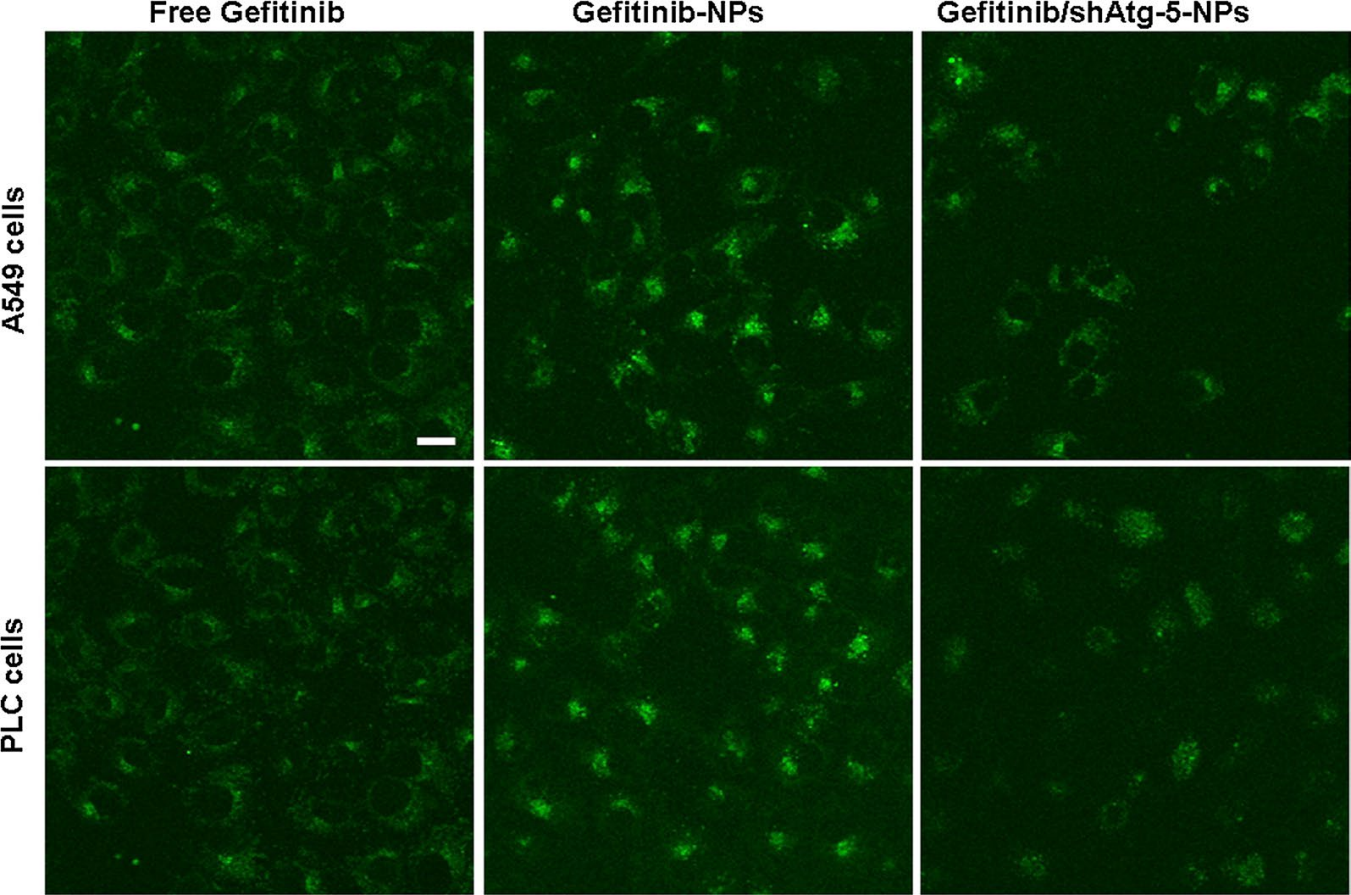
Confocal microscope images of GFP-LC3-transfected A549 cells and PLC cells treated with free gefitinib and gefitinib-loaded NPs. *Scale bar* 100 μm

The results showed that when compared with free gefitinib, the exposure of gefitinib-loaded NPs induced the gathering of bright green fluorescent dots in both cells (Fig. 5), indicating that the internalization of gefitinib-loaded NPs enhanced autophagy effects, and that more GFP-LC3 were transferred to the autophagic membranes and aggregated within the cells. When shAtg-5, which was used to silence the Atg-5 gene, combined with gefitinib to treat the cells, the autophagy was significantly inhibited, as represented by the reduced green fluorescent aggregation of endogenous LC3 in both cell types.

### Cell apoptosis and necrosis

**Fig. 6:**
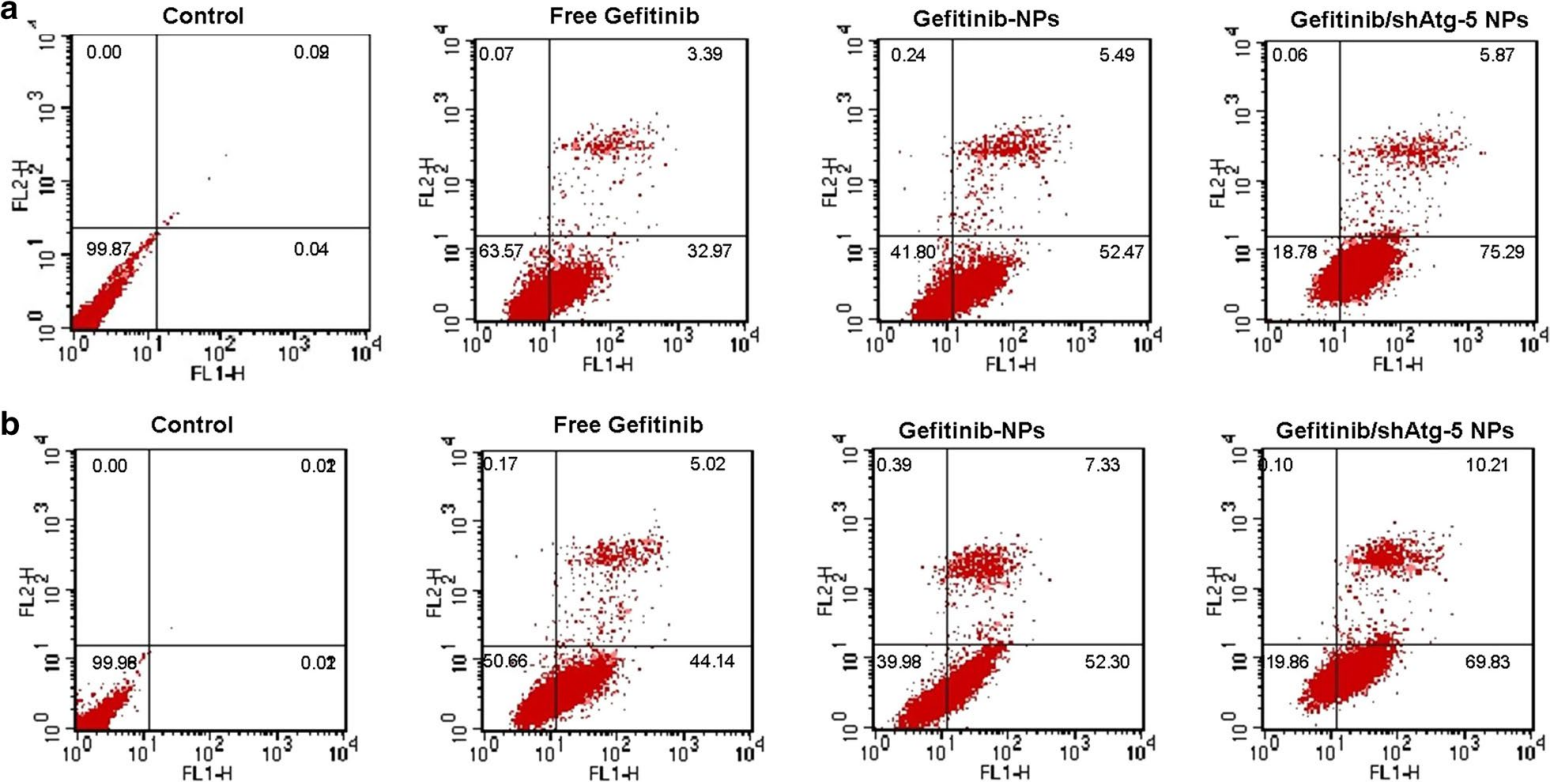
The early apoptosis of A549 cells (**a**) and PLC cells (**b**) treated with free gefitinib, gefitinib NPs, and gefitinib/shAtg-5 NPs

To investigate the synergistic effects of the co-delivery of shAtg-5 and gefitinib on cell apoptosis, an Annexin V-FITC/PI staining assay was performed, and the apoptotic and necrotic cells were quantified by flow cytometry. The results shown in Fig. 6 highlighted that the addition of gefitinib enhanced the apoptosis rates in A549 cells and PLC cells. The ratios of AV-positive and PI-positive cells treated with free gefitinib were 36.36% for A549 cells and 49.16% for PLC cells, respectively. The ratios of AV-positive and PI-positive cells treated with gefitinib-loaded NPs were 57.96% for A549 cells and 59.63% for PLC cells, respectively. With the suppression of the Atg-5 protein in cells obtained by the delivery of shAtg-5 to silence target gene expression via RNA interference, the autophagy effects were significantly reduced and the apoptotic effects were further strengthened. The co-delivery of gefitinib and shAtg-5 encapsulated in NPs not only enhanced the transfection efficacy of shRNA by preventing its degradation from the enzyme and serum through encapsulation, but also induced the greatest cell apoptosis effects through the efficient silencing of the Atg-5 gene. The ratio of AV-positive and PI-positive cells treated with gefitinib/shAtg-5 NPs was 81.16% for A549 cells and 80.04% for PLC cells. It can thus be concluded that autophagy inhibition in the presence of shAtg-5 loaded in NPs had significantly decreased cell viability, and such cell death was associated with apoptosis induction, as revealed by fluorescence-activated cell sorting (FACS) analysis.

### Western blot assay

**Fig. 7:**
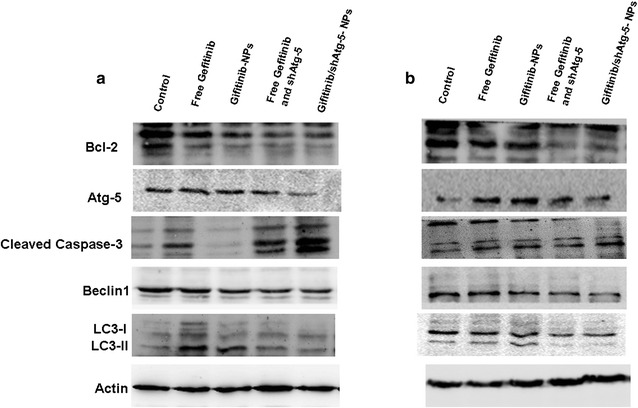
Apoptotic effects of various gefitinib formulations on A549 cells (**a**) and PLC cells (**b**). Western blot analyses of the expression levels of cleaved caspase-3, Bcl-2, Beclin1, Atg-5, and LC3 proteins in A549 cells and PLC cells following treatment

To confirm the relationship between autophagy and cellular apoptosis, we performed western blotting of the protein extracts from the cells to determine the expression changes of some autophagy marker proteins, such as microtubule-associated protein 1 light chain 3 (LC3), Atg-5, and beclin1. In addition, the expressions of apoptosis-related proteins (cleaved caspase-3 and bcl-2) were also examined by western blot. Figure 7 demonstrated that in accordance with the flow cytometry analysis results, the cellular apoptosis effects had also been improved in A549 cells and PLC cells treated with the free drug or drug-loaded NPs. The results showed that the expression of cleaved caspase-3 as the primary marker for cellular apoptosis was upregulated. In the meantime, with the addition of gefitinib or gefitinib-loaded NPs into the cells, autophagy was triggered to promote tumor cell survival by degrading apoptotic mediators. Therefore, the autophagy effects were also strengthened, as represented by the increased ratio of LC3 II to LC3 I. The Atg-5 protein was encoded by the Atg-5 gene, and its function in autophagy was to regulate autophagosome elongation. With the introduction of shAtg-5-loaded NPs, the Atg-5 protein was effectively silenced and the ratio of LC3 II to LC3 I had been significantly decreased in both cells, suggesting that autophagy was inhibited to a greater degree through the efficient suppression of the Atg-5 protein. Compared to the group that was treated with free gefitinib and gefitinib NPs, the co-delivery of gefitinib and shAtg-5 within the NPs induced the highest expression of cleaved caspase-3 in both cells, indicating that the cells’ sensitivity to gefitinib was significantly enhanced with the mediation of Atg-5, ultimately inducing significant cellular apoptosis effects. It also demonstrated that autophagy could adversely affect apoptosis, and that the blockade of autophagy would increase the cells’ sensitivity to apoptotic signals.

### In vivo anti-tumor effects

**Fig. 8:**
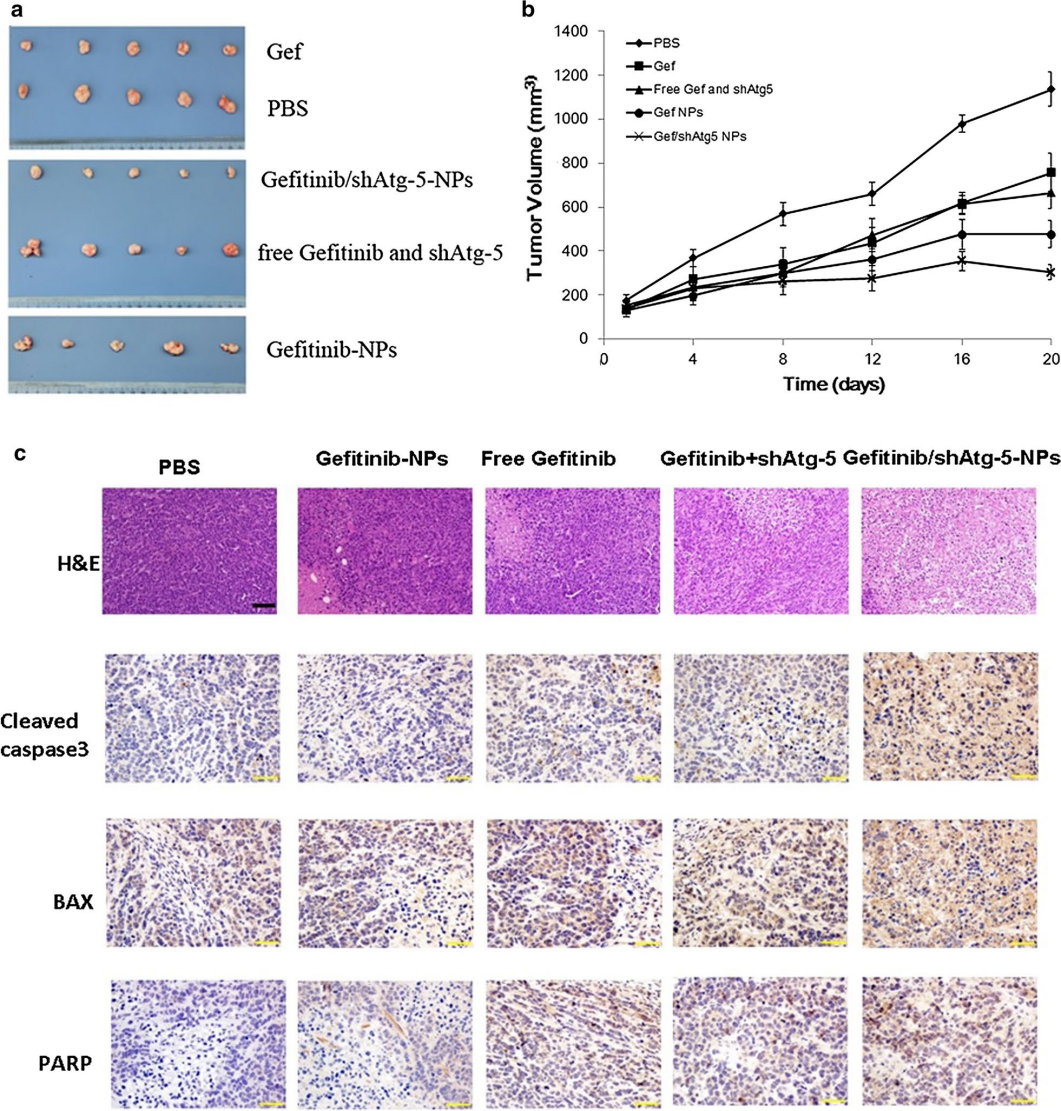
Tumor growth inhibition and histological examination of tumor sections in nude mice bearing PLC tumors following treatment with different formulations. **a** Images of a xenograft tumor model in vivo following treatment with PBS, free gefitinib, free gefitinib and shAtg-5, gefitinib NPs, and gefitinib/shAtg-5 NPs for 3 weeks. **b** Tumor volume curves after the animals were treated by different preparations. The results were expressed as mean ± SD (n = 5). **c** Histological examination of tumor sections (20 days after the first treatment). Nuclei were stained blue, while the extracellular matrix and cytoplasm were stained red via H&E staining. For the immunohistochemistry staining, blue stains showed the nuclei, and brown stains in the cytoplasm indicated protein expression in tumor cells. *Scale bar* for H&E staining: 40 μm; *scale bar* for immunohistochemistry staining: 20 μm

To explore whether the co-delivery of shAtg5 and gefitinib via NPs could facilitate the sensitivity of PLC cells to gefitinib in vivo, we used an ectopic xenograft model to detect this agent’s in vivo anti-tumor effect. The results in Fig. 8 showed that free gefitinib and gefitinib NPs treatment resulted in a mild reduction in tumor weight in PLC tumors, relative to those tumors treated with PBS. In contrast, after treating PLC tumors with gefitinib/shAtg-5 NPs, the resulting apoptosis markedly reduced the tumor weight. These volumetric changes are showcased in Fig. 8a, b, as the gefitinib/shAtg-5-loaded NPs had advantages over free gefitinib and single gefitinib-loaded NPs in inhibiting the growth of solid tumors. To further confirm the tumor-inhibiting effects of gefitinib/shAtg-5 NPs, a histopathological analysis was conducted to exam the extent of damage to the PLC cells. The hematoxylin and eosin (H&E) staining images in Fig. 8c showed that a large number of tumor cells were observed in the PBS group, and that the most extensive tissue necrosis was found in the group treated with gefitinib/shAtg-5 NPs when compared with free gefitinib, free gefitinib and shAtg-5, and gefitinib NPs. Treatment with gefitinib/shAtg-5 NPs induced the highest levels of cell necrosis, cell lysis, and fragmentation, as confirmed by H&E staining. Moreover, the levels of apoptosis-related proteins, such as cleaved caspase-3, bax, and parp, had been significantly increased. These findings revealed that the co-delivery of gefitinib/shAtg-5 in the NPs produced superior anti-cancer effects and significantly inhibited the rapid growth of solid tumors.

## Discussion

Autophagy is a cellular catabolic process in which cytoplasmic target material is transported to the lysosomes for degradation through a wide array of resident acid hydrolases. It is well known that autophagy is critical in material recycling and cell homeostasis maintenance; this process removes damaged proteins and cell organelles in stress states, thus limiting the extent of cellular damage and maintaining cell stability. In tumor therapy, autophagy has been shown to be one of the factors responsible for chemotherapy, radiotherapy, and biological immunotherapy tolerance. Conversely, prolonged and uncontrolled autophagy is also involved in cell death [33].

In our work, we tried to clarify the role of autophagy induced by a free drug and drug-loaded NPs in the induction of apoptosis in cancer cells. It was found that free gefitinib and gefitinib-loaded NPs induced cell death, while the autophagy effects were also simultaneously enhanced to some extent, which indicated that autophagy might have some negative effects on the induction of cell apoptosis. The introduction of shAtg-5 as an inhibitor of autophagy efficiently downregulated the expression of the autophagy-related protein Atg-5; furthermore, and a series of experiments were conducted, which proved that autophagy inhibition occurred, as evidenced by the decreased ratio of LC3 II to LC3 I. Furthermore, the cytotoxicity and apoptosis of cells treated with the co-delivery of shAtg-5 and gefitinib loaded in CS NPs had been significantly enhanced in association with autophagy inhibition. When compared with free gefitinib, gefitinib and shAtg-5, and gefitinib NPs, the IC50 values of gefitinib/shAtg-5 NP-treated A549 and PLC cells within 24 h were lowest. The ratio of AV-positive and PI-positive cells treated with gefitinib/shAtg-5 NPs had increased to 81. 16% for A549 cells and to 80.04% for PLC cells. It is possible that when treating cells with the free drug or with drug-loaded NPs, autophagy was triggered and played a cytoprotective role in response to the toxicity exerted by the drug or drug-loaded NPs, which could provide a lot of nutrients and oxygen to improve tumor cell adaptability and to promote cell survival, thus preventing apoptosis and necrosis. The involvement of shAtg-5 offered specific and long-lasting gene silencing effects, and autophagy was significantly downregulated. More importantly, the cells’ sensitivity to gefitinib was enhanced by increasing the apoptosis of A549 cells and PLC cells, as induced by gefitinib. In addition, NPs protected shAtg-5 through encapsulation while avoiding degradation by enzymes, and they further improved the gene transfection efficiency and accomplished effective gene silencing.

All of these results showed that autophagy as a protective mechanism could be induced in tumor cells with the mediation of gefitinib as an anti-cancer drug; it ultimately reduced cytotoxicity during cell death, enhanced the adaptation of tumor cells to the environment, and it even led to chemotherapy tolerance. Co-delivery of shAtg-5 and gefitinib loaded in CS NPs triggered the apoptosis pathway and enhanced synergistic antitumor effects via autophagy blockade.

## Conclusions

CS NPs prepared by an ion gelation method demonstrated excellent properties such as good drug entrapment, sustained release, smaller average particle size, a low PDI, and a high EE, and it accomplished the efficient co-delivery of shAtg-5 and gefitinib. shAtg-5 was involved as a powerful tool in the silencing of the Atg-5 protein and in the treatment of A549 cells and PLC cells, as shAtg-5 induced autophagy inhibition, while further increasing cell inhibition and apoptosis when treated with a combination of shAtg-5 and gefitinib. This finding suggested that when compared with treatment with gefitinib alone, the co-delivery of gefitinib and shAtg-5 encapsulated within the NPs greatly contributed to gefitinib-induced apoptosis among A549 cells and PLC cells by significantly inhibiting autophagy and inducing cellular apoptosis. Taken together, the co-delivery of shAtg-5 and gefitinib loaded in the CS NPs enhanced the synergistic antitumor effects via autophagy blockade.

## Abbreviations

CS: chitosan
NPs: nanoparticles
TPP: sodium tripolyphosphate
EGFP: enhanced green fluorescent protein

## References

[1] Sotiropoulou PA, Christodoulou MS, Silvani A (2014). Chemical approaches to targeting drug resistance in cancer stem cells. Drug Discov Today.

[2] Kathawala RJ, Gupta P, Ashby CR, Chen ZS (2015). The modulation of ABC transporter-mediated multidrug resistance in cancer: a review of the past decade. Drug Resist Updat.

[3] Kibria G, Hatakeyama H, Harashima H (2014). Cancer multidrug resistance: mechanisms involved and strategies for circumvention using a drug delivery system. Arch Pharm Res.

[4] Chen N, Debnath J (2010). Autophagy and tumorigenesis. FEBS Lett.

[5] Rao S, Yang H, Penninger JM, Kroemer G (2014). Autophagy in non-small cell lung carcinogenesis: a positive regulator of antitumor immunosurveillance. Autophagy.

[6] Mukhtar E, Adhami VM, Khan N, Mukhtar H (2012). Apoptosis and autophagy induction as mechanism of cancer prevention by naturally occurring dietary agents. Curr Drug Targets.

[7] Chaabane W, User SD, El-Gazzah M (2013). Autophagy, apoptosis, mitoptosis and necrosis: interdependence between those pathways and effects on cancer. Arch Immunol Ther Exp.

[8] Xie Y, Murray-Stewart T, Wang Y (2017). Self-immolative nanoparticles for simultaneous delivery of microRNA and targeting of polyamine metabolism in combination cancer therapy. J Control Release.

[9] Conde J, Oliva N, Zhang Y, Artzi N (2016). Local triple-combination therapy results in tumour regression and prevents recurrence in a colon cancer model. Nat Mater.

[10] Duan S, Yang Y, Zhang C, Zhao N, Xu FJ (2016). NIR-responsive polycationic gatekeeper-cloaked hetero-nanoparticles for multimodal imaging-guided triple-combination therapy of cancer. Small.

[11] Elgogary A, Xu Q, Poore B, Alt J (2016). Combination therapy with BPTES nanoparticles and metformin targets the metabolic heterogeneity of pancreatic cancer. Proc Natl Acad Sci USA.

[12] Gilam A, Conde J, Weissglas-Volkov D, Oliva N, Friedman E, Artzi N, Shomron N (2016). Local microRNA delivery targets Palladin and prevents metastatic breast cancer. Nat Commun.

[13] Kong F, Zhang H, Qu X (2016). Gold nanorods, DNA origami, and porous silicon nanoparticle-functionalized biocompatible double emulsion for versatile targeted therapeutics and antibody combination therapy. Adv Mater.

[14] Conde J, Bao C, Tan Y (2015). Dual targeted immunotherapy via in vivo delivery of biohybrid RNAi-peptide nanoparticles to tumour-associated macrophages and cancer cells. Adv Funct Mater.

[15] Maeda H, Nakamura H, Fang J (2013). The EPR effect for macromolecular drug delivery to solid tumors: improvement of tumor uptake, lowering of systemic toxicity, and distinct tumor imaging in vivo. Adv Drug Deliv Rev.

[16] Panzarini E, Dini L (2014). Nanomaterial-induced autophagy: a new reversal MDR tool in cancer therapy?. Mol Pharm.

[17] Bazak R, Houri M, El Achy S (2015). Cancer active targeting by nanoparticles: a comprehensive review of literature. J Cancer Res Clin Oncol.

[18] Gao Z, Zhang L, Sun Y (2012). Nanotechnology applied to overcome tumor drug resistance. J Control Release.

[19] Liu Y, Wang Y, Zhang C (2014). Core–shell nanoparticles based on pullulan and poly(β-amino) ester for hepatoma-targeted codelivery of gene and chemotherapy agent. ACS Appl Mater Interfaces.

[20] Guan X, Li Y, Jiao Z (2015). Codelivery of antitumor drug and gene by a pH-sensitive charge-conversion system. ACS Appl Mater Interfaces.

[21] Tsouris V, Joo MK, Kim SH (2014). Nano carriers that enable co-delivery of chemotherapy and RNAi agents for treatment of drug-resistant cancers. Biotechnol Adv.

[22] Dong D, Gao W, Liu Y, Qi XR (2015). Therapeutic potential of targeted multifunctional nanocomplex co-delivery of siRNA and low-dose doxorubicin in breast cancer. Cancer Lett.

[23] Song H, Su C, Cui W, et al. Folic acid-chitosan conjugated nanoparticles for improving tumor-targeted drug delivery. Biomed Res Int. 2013:723158. 10.1155/2013/723158 PMC3825055 24282819

[24] DeBiasio R, Bright GR, Ernst LA, Waggoner AS, Taylor DL (1987). Five-parameter fluorescence imaging: wound healing of living Swiss 3T3 cells. J Cell Biol.

[25] Brauns SC, Milne P, Naudé R, Van de Venter M (2004). Selected cyclic dipeptides inhibit cancer cell growth and induce apoptosis in HT-29 colon cancer cells. Anticancer Res.

[26] Su C, Li H, Shi Y (2014). Carboxymethyl-β-cyclodextrin conjugated nanoparticles facilitate therapy for folate receptor-positive tumor with the mediation of folic acid. Int J Pharm.

[27] Zhao L, Su R, Cui W (2014). Preparation of biocompatible heat-labile enterotoxin subunit B-bovine serum albumin nanoparticles for improving tumor-targeted drug delivery via heat-labile enterotoxin subunit B mediation. Int J Nanomedicine.

[28] Shukla SK, Mishra AK, Arotiba OA, Mamba BB (2013). Chitosan-based nanomaterials: a state-of-the-art review. Int J Biol Macromol.

[29] Yang Y, Wang S, Wang Y (2014). Advances in self-assembled chitosan nanomaterials for drug delivery. Biotechnol Adv.

[30] Garcia-Fuentes M, Alonso MJ (2012). Chitosan-based drug nanocarriers: where do we stand?. J Control Release.

[31] Paiva D, Ivanova G, Pereira Mdo C, Rocha S (2013). Chitosan conjugates for DNA delivery. Phys Chem Chem Phys.

[32] Yhee JY, Song S, Lee SJ (2015). Cancer-targeted MDR-1 siRNA delivery using self-cross-linked glycol chitosan nanoparticles to overcome drug resistance. J Control Release.

[33] Eisenberg-Lerner A, Bialik S, Simon HU, Kimchi A (2009). Life and death partners: apoptosis, autophagy and the cross-talk between them. Cell Death Differ.

